# Supplementary material for: Human Cognitive Neuroscience as It Is Taught
Source: Front Psychol. 2020 Nov 24;11:587922. doi: 10.3389/fpsyg.2020.587922 (PMC7732510; doi:10.3389/fpsyg.2020.587922)
Supplement: Supplementary file 1 [file Table_1.docx]

Appendix:

## **Survey questions and multiple-choice answers**

The methods-related questions of the survey, grouped into categories, are listed below. Every question also had a “No idea” option, which is not listed here.

## Statistics:

The correlation coefficient between two signals describes

- whether one signal is larger than the other
- the linear relationship between the signals
- whether one signal is the derivative of the other
- the sound quality of the signals

In statistics, a power analysis can be used to determine

- whether an analysis is sensitive enough to detect an effect of a certain size
- how much computer memory you need to perform an analysis
- whether an effect is statistically significant
- how strong your signal is

The statistical "multiple comparisons problem" refers to

- the decrease of statistical power with large data sets
- the increased uncertainty in your result with too few samples
- the increase of computing power required to perform multiple statistical tests
- the increased likelihood of false positives if one performs multiple independent tests

## Signal Analysis

The “signal to noise ratio” (SNR) refers to

- a measure for the amplitude of the desired part of the signal divided by a similar measure for the undesired part of the signal
- a measure for the amplitude of the undesired part of the signal divided by a similar measure for the desired part of the signal
- the ratio between two random signals
- a statistical comparison between a structured and a random model

If you have two signals with frequencies f1 and f2, then the sum of these signals has peaks in the frequency spectrum at which frequency or frequencies?

- f1+f2 and f1-f2
- f1 and f2
- f1*f2
- (f1+f2)/2

A Fourier analysis is

- a method to decompose signals into shorter sequences
- a method to determine the four most significant components of a signal
- a method to decompose signals into complex polynomials
- a method to decompose signals into sine and cosine functions

The sine of an angle in a right-angled triangle is equal to

- opposite divided by adjacent leg
- adjacent divided by opposite leg
- opposite leg divided by hypotenuse
- adjacent leg divided by hypotenuse

A complex number is

- a number that cannot be expressed as the ratio of two integers
- a number that is difficult to keep in working memory
- a number that involves the square root of -1
- a number with more than 10 digits

## Calculus

If function f is the integral of the function g, then

- f divided by g is unity
- the derivative of f is g
- the integral of f is g
- f and g are orthogonal

The derivative of "2 times x-squared" is:

- 4 times x
- 2 times x-squared
- 4 times x-cubed
- 2 times x

What is the solution to 3*x – 2 = 7?

- 1
- 3
- 5
- 12

## Linear Algebra

What is the (scalar) product of the two vectors [1 2] and [3 4]?

- 4
- 21
- 10
- 11

Which of these pairs of vectors are orthogonal?

- [1 2], [1 2]
- [0 1], [0 1]
- [1 2], [3 4]
- [1 -2], [2 1]

## Programming

An integer number is

- a number that can be written without a fractional component
- a number that cannot be divided
- a prime number smaller than 100
- a positive number

In software programming, a “for loop” is a statement that

- circumvents an error message
- asks for further information
- allows a piece of code to be repeatedly executed
- lets the program return to a previous statement

“Linux” is

- the first scientific computer game
- a powerful text editor
- a software for scientific data analysis
- an open source computer operating system

## Physics

Ohm’s law, i.e. the relationship between voltage and current in an electric circuit, states that

- Voltage = Resistance / Current
- Current = Resistance * Voltage
- Current = Voltage / Resistance
- Current = Voltage - Resistance

The electric field is

- the gradient of the electric potential
- the integral of the electric potential
- the square of the electric potential
- the distribution of the electric potential visualised as arrows

## B) Components of a skills-oriented training programme for cognitive neuroscientists.

The first column indicates the category of topics that could be taught as separate modules. The second columns presents some examples from different domains of neuroimaging for which these categories are relevant. The third columns lists possible specific topics that could be taught in these modules.

| **Category** | **Example applications in neuroimaging** | **Specific topics** |
| --- | --- | --- |
| Linear Algebra | General:   - Artefact correction, filtering - Optimisation - Statistics - Computational modelling   (f)MRI:   - First and second level statistics   EEG/MEG:   - Forward and inverse problem - Source estimation | - Vectors and matrices - Vector and matrix multiplication, matrix algebra - Vector spaces, matrix rank - Vector norms - Linear (in)dependence, orthogonality, orthonormality - Matrix decompositions (PCA, SVD) - Matrix (pseudo)inversion - General linear model, over-/under-determined problems - Ill-conditioned matrices, regularisation - Matrix norms |
| Time and frequency domain analysis | General:   - Filtering - Computational modelling   (f)MRI:   - k-space and image processing - Convolution with HRF - Connectivity   EEG/MEG:   - Brain oscillations - Connectivity | - Periodic signals, sine and cosine - Complex numbers and their relationship to periodic signals (Euler’s formula) - Fourier decomposition - Time-frequency decomposition (wavelets and Hilbert transform) - Convolution and filtering and their relationship in time and frequency domain - Autocorrelation and coherence - Measures of phase synchrony - Auto-regressive models (uni- and multi-variate) |
| Calculus | General:   - Structural equation modelling - Optimisation - Computational modelling   (f)MRI:   - DCM   EEG/MEG:   - Forward and inverse problem - DCM - Coupled oscillators | - Derivatives and integrals in time and frequency domain - Differential equations - Structural equation modelling |
| Physical principles | (f)MRI:   - Health and safety in scanner - Signal artefacts and distortions, eddy currents - Image acquisition sequences   EEG/MEG:   - EEG vs MEG, sensitivity of sensor types - Sources of artefacts - Head modelling (forward/inverse problem) | - Electric charges and currents (resistance/conductivity, Ohm’s law, capacitance) - Static electric and magnetic fields and their sources (Coulomb’s and Ampere’s law) - Properties of static electric and magnetic fields (Gauss’ and Biot-Savart’s law) - Electromagnetic induction (Faraday’s law) - Relationship between electric and magnetic fields, Maxwell’s equations - Electromagnetic waves |
| Scientific Computing | General, (f)MRI, EEG/MEG:   - Develop new analysis pipelines - Modify and debug existing analysis pipelines - Understand and reproduce previous analyses - Communicate with software developers and colleagues | - Assigning and manipulating variables - Vectors, matrices, arrays etc. - Basic built-in functions (average, sine/cosine, etc.) and linear algebra - Visualisation - Reading and writing data (numeric, text) - For and while loops - Conditional statements - Functions - Choosing and using a text editor or integrated development environment (IDE) - Organising and documenting code - Testing and debugging code - Optimising code (parallel computing) - Publicising and sharing code |
